# Winter and spring climatic conditions influence timing and synchrony of calving in reindeer

**DOI:** 10.1371/journal.pone.0195603

**Published:** 2018-04-25

**Authors:** Amélie Paoli, Robert B. Weladji, Øystein Holand, Jouko Kumpula

**Affiliations:** 1 Department of Biology, Concordia University, Montreal, Quebec, Canada; 2 Department of Animal and Aquacultural Sciences, Norwegian University of Life Sciences, Ås, Norway; 3 Natural Resources Institute of Finland, Reindeer Research Station, Kaamanen, Finland; Université de Sherbrooke, CANADA

## Abstract

In a context of climate change, a mismatch has been shown to occur between some species’ reproductive phenology and their environment. So far, few studies have either documented temporal trends in calving phenology or assessed which climatic variables influence the calving phenology in ungulate species, yet the phenology of ungulates’ births affects offspring survival and population’s recruitment rate. Using a long-term dataset (45 years) of birth dates of a semi-domesticated reindeer population in Kaamanen, North Finland, we show that calving season has advanced by ~ 7 days between 1970 and 2016. Advanced birth dates were associated with lower precipitation and a reduced snow cover in April and warmer temperatures in April-May. Improved females’ physical condition in late gestation due to warmer temperatures in April-May and reduced snow conditions in April probably accounted for such advance in calving date. On the other hand, a lengthening of the calving season was reported following a warmer temperature in January, a higher number of days when mean temperature exceeds 0°C in October-November and a decreasing snow cover from October to November. By affecting the inter-individual heterogeneity in the plastic response of females’ calving date to better climatic conditions in fall and winter, climatic variability contributed to weaken the calving synchrony in this herd. Whether variability in climatic conditions form environmental cues for the adaptation of calving phenology by females to climate change is however uncertain, but it is likely. As such this study enhances our understanding on how reproductive phenology of ungulate species would be affected by climate change.

## Introduction

Reproductive synchrony is the tendency of individuals to carry out parts of their reproductive cycle at the same time as other members of the population [1,2]. In natural populations of either plant or animal species, reproductive synchrony is the result of natural selection when a reproductive advantage (e.g. reduced predation) is conferred to individuals breeding in a synchronous pattern [3]. Being the main ultimate factor of reproductive synchrony, offspring survival can be affected by multiple factors, such as climate, predation and sociobiological. Reproductive synchrony as a mean to reduce predation is explained by several hypotheses: first, the ‘saturation hypothesis’ suggests that predators will be overwhelmed if all young are born in a brief period [4,5]; second, adults breeding synchronously could use vigilance to detect predators more efficiently; third [3,6], the ‘confusion hypothesis’ states that a high number of young in a group will decrease the predator’s capacity to pursue a specific target [4,7]. The wildebeest (*Connochaetes taurinus*) provides the best known example of synchronized calving, where predation pressure by the hyena (*Crocuta crocuta*) has promoted a short birth peak and an aggregated over a dispersed spatial distribution of individuals in order to ensure the survival of the young [5,8].

Several studies on northern ungulates have shown that climatic variability contributed more than predation in constraining timing and synchrony of births: bighorn sheep (*Ovis canadensis*) [9], caribou (*Rangifer tarandus*) [10], Dall’s sheep (*Ovis dalli*) [7,11], reindeer (*Rangifer tarandus*) [12] and roe deer (*Capreolus capreolus*) [13]. In temperate and subarctic climates, a marked seasonality in forage availability has been shown to strongly influence both perinatal and neonatal mortality of ungulates [9,14] and thus explains variation in synchrony of ungulates’ births [15]. Individuals born outside the optimal period for births will have lower probabilities to survive [14,15] because (1) they will be more vulnerable to predation by bears, golden eagles and other predators [16,17], (2) if born too early, mothers can be in negative energy balance therefore producing a low quality milk [11,18] and (3) if born too late, young will be more susceptible to insect harassment and summer heat [19,20] and will not accumulate enough resources; ultimately reducing their survival rate during summer season, and during their first winter thereafter [9]. The calving phenology resulting from thousands of years of evolution is thus expected to reflect the species’ adaptation to its environment.

In a context of the worldwide global warming recorded the last decades, a mistiming has been shown to occur between species’ reproductive phenology and their environment leading to a decrease in their recruitment rate: in great tits (*Parus major*) [21], several species of birds [22], caribou [23], reindeer [24], squirrel (*Urocitellus columbianus*) [25]. Determining the relationship between reproductive tactics and a species environment and understanding the role of phenotypic plasticity on reproductive traits are therefore crucial to predict how climate change will affect species’ viability. Ungulates with highly synchronized births in particular are of primary concern because they are more susceptible to climatic variation than asynchronously breeding ungulates that are better adapted to large changes in climate [26]. *Rangifer* species (including both caribou and reindeer) in this context is certainly the most vulnerable species since: (1) this is one of the two ungulate species to have successfully colonized the unpredictable and austere arctic environment and (2) *Rangifer* species has been shown to produce 80–90% of their calves within a 10-day period and complete the calving season within 4–5 weeks [12,27,28]. Moreover, herding and hunting of *Rangifer* allowed northern peoples in the Arctic Circle to survive in a harsh and austere environment, and constitute the cultural and socioeconomic pillar of these cultures [29]. Surprisingly, reindeer has not received enough attention with respect to the global climate change debate (but see [29–32]). Our aim here is thus to investigate how the reindeers’ calving phenology is affected by climatic variability by using a long term dataset of birth dates recorded since 1970 in a semi-domesticated reindeer population in Northern Finland. In this study, the calving phenology will be assessed using both the date when births occur and the length/synchrony of the births season.

Since births in ungulates have been reported to occur later following winters with colder temperatures [33], higher amounts of snowfall [28,34] and deep snow cover [35], females are expected to present a plastic response in calving date according to the severity of winter and consequently the calving season is expected to occur earlier with a reduced snow cover, and an overall warmer and wetter climate as predicted over Northern Hemisphere [36,37]. Such climatic changes, by contributing to the lengthening of the vegetative growing season [36,38], would release selective pressure to having births highly concentrated in time to match the forage resources [4] and a lengthening of the calving season would result. The aims in the present study are to: (1) quantify rates of temporal change of reindeer reproductive phenology (date and length) and of climatic variables for our study site and (2) assess whether phenological changes in reindeer reproduction can be explained by the variation in local climatic condition.

## Material and methods

### Study area and reindeer population

The data collected come from the Kutuharju field reindeer research station in Kaamanen, northern Finland (69°N, 27°E). The area is characterized by open birch *Betula spp*. and pine *Pinus sylvestris* forests with many bogs and lakes and the landscape varies between 185–370 m above the sea level. We studied a semi-domesticated reindeer population constituted of about 100 animals every year. All animals were marked with ear tags from birth, allowing their age to be known, while being individually recognizable thanks to the long term book-keeping of the herd demography. Reindeer are free ranging most of the year, excluding the calving period. In summer and during the rut, reindeer use two large fenced enclosures, the north-west section (Lauluvaara ~ 13.8 km^2^) and the south-east section (Sinioaivi ~ 15 km^2^). After the breeding season in late October, the animals are gathered and taken to a winter grazing area (15 km^2^) where they can graze freely on natural pastures. Only in late winter and especially after harsh winters, animals receive in addition supplementary feed (pellets and hay). In late April, females are gathered into a calving enclosure (approximately 0.5 km^2^) where newborn calves are captured, weighed, sexed and marked with ear tags [39]. The enclosure is surveyed daily, so that calving date is known for all individuals and has been recorded since 1970.

### Calving season

All calendar dates were converted into Julian days since 1 January for analysis purposes. Assuming that the calving dates follow a bell curve, the synchrony—or length—of the calving season (when 95% of births occurred) was estimated as the width of the 95% confidence interval around the peak date of each period, a function of the within-year variance used by Loe et al. [40] and calculated as two times twice the standard deviation (2 × 2*σ*) of the whole calving season in any given year. In total, 45 years of data were available for both the calving date and the length of the calving season.

### Population variables

**Fig 1:**
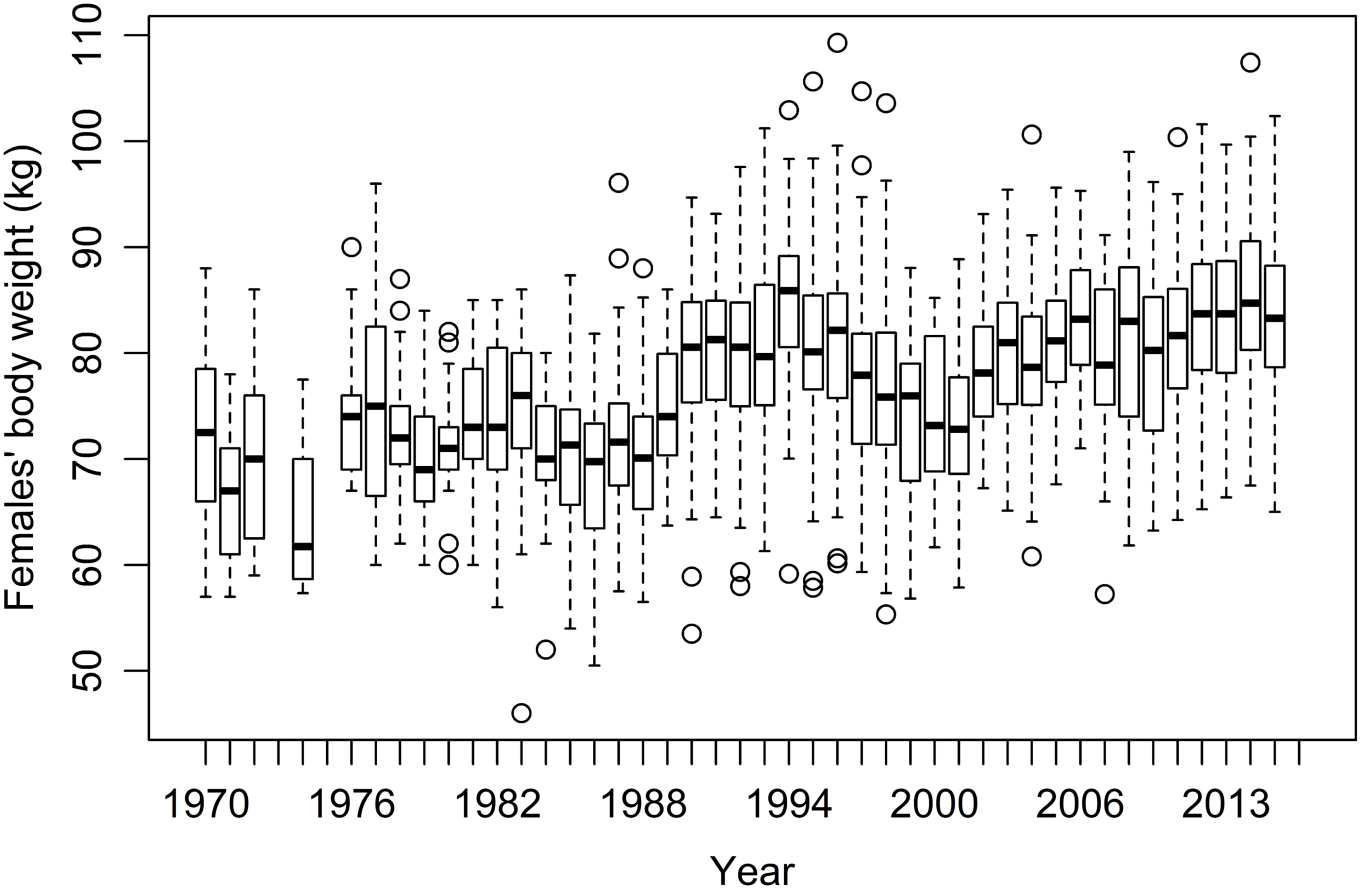
Within and between year variability in adult females’ body weight of the Kutuharju field reindeer research station herd between 1970 and 2016 in northern Finland. Each female’s body weight per year was calculated as the average value of the recorded body weights for that female from June to December the precedent calendar year and from January to May the same calendar year as the female’s calving season.

Being a research herd, several experiments have been conducted on this reindeer population for different purposes. Thanks to the book-keeping of the herd, the identity of the animals involved in any experiment was known. Experimental animals were excluded from our analyses when: (1) males or females isolated for experimental purpose could have been subjected to other factors and do not reflect the overall trend of the herd and (2) an artificial feeding could have buffered climatic effects on females’ body condition and therefore on calving date. Indeed the calving date has been shown to be strongly influenced by female’s body weight at different periods of the year [35,41–45]. Because artificial feeding in 2009 was done more than would be expected in a normal year, we excluded the data for year 2009 from the analyses since earlier calving dates could just be the result of females being heavier that particular year, independently of climatic conditions. Given the great variability within and between years in females’ body weight (see Fig 1), we believe that regular level of supplemental feeding alone could not buffer climatic effects by keeping up females’ body weight at a stable level, hence our decision to remove year 2009.

To control for the effect of proportion of males on breeding time and thereafter on birth dates [44,46,47], the proportion of males was estimated as the number of males divided by the number of females present in a specific enclosure during the breeding season. Between 1996 and 2011 (except 1998), the herd was separated in the two large enclosures, Sinioivi and Lauluvaara and consequently the proportion of males was estimated per enclosure for those years. Using the identity of the females present in each enclosure, the calving date of a specific female was related to the proportion of males estimated in that enclosure the past breeding season. The effect of proportion of males on breeding time and consequently on calving date [44,46,47] was thus controlled for in the analyses.

### Climatic data

#### Local weather variables

Local climatic data (daily recorded values for temperature, precipitation and snow cover) from 1970 to 2016 have been obtained from three different weather stations (Utsjoki, Ivalo airport and Nellim) in north Finland (68°N, 27°E) from the Finnish Meteorological Institute, Helsinki, Finland. The weighted mean by the distance from the weather station to our study site was used to estimate the local weather at our study site with as much reliability as possible. A Great Circle longitude-latitude calculations tool (http://www.cpearson.com/excel/LatLong.aspx) was used to assess precisely the distance between our study site and each of the weather stations, based on the GPS coordinates of the two locations. All the weather variables were considered on a monthly basis. Monthly average temperature was the average of the mean daily temperatures recorded over a month whereas the sum was used for precipitation and snow depth. Precipitation includes rain and/or snow depending on the temperature. Snow depth index (SDI) was calculated as the cumulative sum of daily snow depths on the 15^th^ day in each month. Moreover, the following temperature parameters for each month were used to reflect the climatic variation: number of days when the mean temperature exceeds 0°C and 5°C [48] and number of days when the mean temperature goes below -10°C. All the weather variables used in the analyses for calving date and synchrony and the references justifying their use are summarized in Table 1. The temporal trend was assessed using linear models with *Year* considered as a continuous variable and entered as a fixed-effect factor in the models.

**Table 1:** Summary of all the weather variables used to analyze the influence of climatic variability on both the calving date and calving synchrony of a semi-domesticated reindeer population for the study area of the Kutuharju field reindeer research station in Kaamanen, northern Finland (69°N, 27°E).

	Local weather variables					
Month	Temperature				Precipitation	Snow cover
	Mean T°(°C)	Number of days when mean T° >		Number of days when mean T° <	Sum (mm)	Snow depth index—SDI (mm)
		0°C	5°C	-10°C		
**January**	X	X			X	X	X
**February**	X	X		X	X	X
**March**	X	X		X	X	X
**April**	X	X	X	X	X	X
**May**	X	X	X		X	X
**June**	X		X		X	
**July**	X				X	
**August**	X		X		X	
**September**	X	X	X		X	
**October**	X	X	X	X	X	X
**November**	X	X	X	X	X	X
**December**	X	X		X	X	X
**References**	[33,45]	[48]	[48]		[49]	[28,33–35,50]

The availability of each weather variable depending on the month is indicated by an “X”. The significant influence of each weather variable on parturition date for different ungulate species is referred in the last line.

### Statistical analyses

The following statements apply to the analyses for both calving date and calving synchrony. Since phenological variation in calving period (timing and synchrony) could be independently influenced by the previous year’s environmental conditions and conditions in the beginning of current year, we performed models using current year calving data (*t*), and climatic data for both current year from January to May (*t*) and precedent calendar year (*t* − 1) from June to December. Calving dates and calving synchrony were used as response variables in the analyses. We centered and standardized all the predictor variables considered (mean = 0, SD = 1) to be on a comparable scale and assessed for multicollinearity among them using the Variation Inflation Factor (VIF). Predictor variables with VIF smaller than five were kept in the model [51]. If several consecutive months of the same weather variable significantly influenced one trait of the calving phenology (timing or synchrony) when considered separately, the mean (for temperature) or the cumulative sum (for precipitation and SDI) was calculated for the entire period. For example, if mean temperature in April and in May significantly influenced calving date when considered separately, then the mean temperature for the period from April to May was instead used in the model in order to avoid multicollinearity.

Before performing a model selection to identify which variables best explained variation in calving phenology (date and length separately), we assessed the change over time of the reindeer calving phenology using two models both with *Year*, the predictor variable considered as a continuous fixed-effect parameter in the models. The first model, had calving date as response variable and we used a Linear Mixed-effects Model (LMM) with *Year* and individual identity included as random factors; while the second model had calving synchrony as response variable and a Linear Model (LM) was used. These two models were not subject to model selection. A model selection was then performed to find combinations from all the explanatory variables used providing the most probable models to explain calving phenology and was based both on the Akaike Information Criterion, corrected for small sample size (AICc) and Akaike weights (AICc weights) to compare the relative performance of the models tested [52,53]. The delta AICc (Δ_*i*_) was calculated to provide a measure of each model relative to the best model (with the lowest AICc value). All models within a ΔAICc of 2 units were retained as competing models since a substantial evidence was given to the model if Δ_*i*_ < 2 [53]. To account for model selection uncertainty and if more than one model were retained as best models in explaining the data then the estimates of the coefficients of parameters in all models with ΔAICc < 2 were averaged, following the model averaging approach [53–56]. We reported the effect of each predictor variable on the response variable considered with model-averaged parameter estimates, as well as their 95% confidence intervals based on our entire list of candidate models. These estimates are weighted based on the relative importance of the models (given by the AICc weights) containing those parameters and only the ‘conditional averages’ were reported, i.e. the averages over the models where the parameters appeared. The variables included in the competing models were considered important if their 95% CIs excluded 0 and only the important variables were further discussed. Since our predictor variables were beforehand centred and standardized, we could directly interpret their main effects even when involved in interactions and thus avoided the potential misinterpretation of main effects between models with and without the interaction term [55,57,58]. Analyses were performed in R 3.3.0 [59].

#### Calving date

The calving dates data was analysed using Linear Mixed-effects Models (LMMs), by running the lmer-function in the R package lme4 ([60], <www.r-project.org>), and with individual identity and year of study being included in the models as random effects to control for repeated measures [61,62]. *Year* included as a random effect also allows accounting for between-year variations. In addition, as female age [18,28,63], female body weight [42,43,45,64] and proportion of males [44,46,47] are known to influence the calving date, their respective effect was controlled for in the models. Since in reindeer factors linked to maternal condition interact with each other [65] so that older individuals tend to be heavier, we used a female body condition index (BCI) so that (1) effects of female body weight controlling for age be taken into account and (2) multicollinearity between these two highly correlated variables be avoided. This body condition index was estimated by a measure of female body weight the year preceding the calving season after the effect of age is controlled—the age-specific residual body mass (see [66,67]). This age-specific residual body mass was calculated by subtracting from each female’s body weight the average body weight of all females of the same age. These population terms were included in every model and formed what we call the “basic model”, i.e.: *Calving date* ~ *♀ BCI* + *PM* + (1|*ID*) + (1|*Year*), with *BCI* the body condition index of females, *PM* the proportion of males, *ID* the individual identity of the mother, and *Year* the year of management. The terms (1|*ID*) and (1|*Year*) meant that they were included as random factors in the models.

To assess which local weather variables over different months best explained variation in the calving date, the weather variables presented in Table 1 were added to the basic model. As physical condition of females can also be influenced by weather variables, the interactions between BCI and weather variables were also tested in the models. The effect size of each predictor variable was estimated by the parameter estimates from the selected model using the restricted maximum likelihood estimates as recommended for mixed effect models [68] whereas the AICc values were calculated using the maximum likelihood methods [52]. Once the most probable models to explain variation in calving date were selected, we then assessed how the calving date was affected by the most important variables by looking at the sign of their conditional averaged slope values extracted from the entire list of our competing models. The conditional *R*^2^ values were calculated to indicate the proportion of variance explained by both the fixed and random factors of the best-fitting models.

#### Calving synchrony

As the calving synchrony (length of the calving season) was estimated annually, linear models were used. The predictor variables used were the same weather variables described in Table 1. Because calving synchrony is estimated annually for the entire population and to control for the effects of population variables (BCI of females and proportion of males) on calving synchrony, an average value of the BCI of all the females per year, as well as an average value of the proportion of males par year was calculated. Our “basic model” for calving synchrony was thus as follow: *Calving synchrony* ~ *♀ BCI* + *PM*, with *BCI* the body condition index of females and *PM* the proportion of males. To assess which weather variable over different months best explained variation in the calving synchrony, the weather variables presented in Table 1 were added to this basic model. As physical condition of females can also be influenced by weather variables, the interactions between BCI and weather variables were also assessed in the models. How the calving synchrony was affected by a weather variable was assessed with the conditional averaged slope values extracted from the most important variables of our competing models. Adjusted *R*^2^ values were calculated to indicate the proportion of variance explained by the best-fitting models.

## Results

After the exclusion of the birth dates coming from artificially fed females for specific experiments, of females with unknown body weight (as we wanted to correct for the mothers’ physical condition influence on calving date), and of calving dates with unknown related proportion of males, 2,137 birth dates in total were available over 45 years (minus years 1973, 1975 and 2009 because not enough data were available) for a total of 482 mothers, corresponding on average to 50 births per year. The mean calving date was 19-May and the average length of the calving season was 25 days.

### Temporal trends in calving season

**Fig 2:**
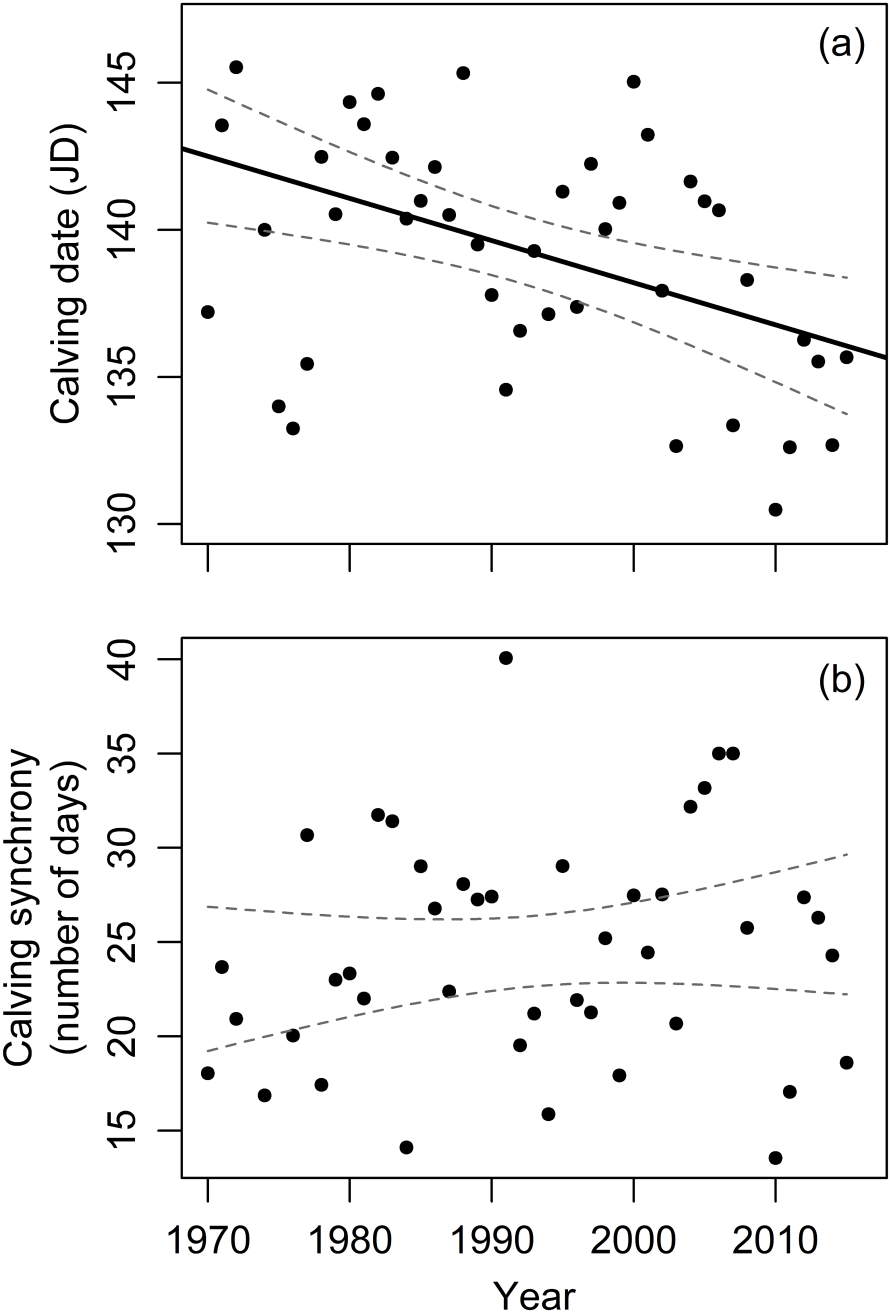
Variation of (a) mean calving date and (b) calving synchrony of a semi-domesticated reindeer population between 1970 and 2016 in Finnish Lapland. The fitted line and the 95% confidence interval band are provided.

Between 1970 and 2016, calving date significantly advanced by an estimated 0.15 (± 0.04 SE) days per year [95% CI = (-0.24, -0.07); Fig 2a]. Across the 45-year study period, calving dates in female reindeer were estimated to have advanced by 6.8 days (Fig 2a). There was a tendency for the duration of the calving season to lengthen over time, i.e. a tendency for calving synchrony to weaken along the study period (*b* = 0.06 ± 0.07 SE; Fig 2b) but this temporal trend was not statistically significant [95% CI = (-0.08, 0.21)].

### Temporal trends in climatic data

Among all the weather variables used in this study and described in Table 1, 16 significant changes over time out of 55 were found (see Table 2). The most noticeable changes being (1) a warming trend from April to May and from August to November, mainly triggered by an increasing number of days when temperature exceeds 0°C in April, an increasing number of days when mean temperature exceeds 5°C in April and in May, and a decreasing number of days when mean temperature goes below -10°C in November; (2) a reduced snow cover from December to February as well as in May and October characterized by a decreasing SDI and (3) an increasing amount of precipitation in May.

**Table 2:** Parameter estimates (with SE) for the linear models with the year of management included as a covariate to assess the temporal trends in all the weather variables for the Kutuharju field reindeer research station, northern Finland.

Weather variables		Estimate	SE	t-value	P	Total change over the study period
**Mean temperature (°C)**	*April*	0.066	0.019	3.44	< 0.01	+ 3.1°C
	*May*	0.046	0.016	2.89	< 0.01	+ 2.1°C
	*August*	0.028	0.014	2.04	< 0.05	+ 1.3°C
	*September*	0.051	0.015	3.34	< 0.01	+ 2.3°C
	*October*	0.047	0.023	2.02	< 0.05	+ 2.2°C
	*November*	0.09	0.033	2.75	< 0.01	+ 4.1°C
**Number of days when mean T° > 0°C**	*April*	0.19	0.058	3.30	< 0.01	+ 8.9 days
**Number of days when mean T° > 5°C**	*April*	0.037	0.017	2.16	< 0.05	+ 1.7 days
	*May*	0.15	0.056	2.71	< 0.01	+ 7.0 days
**Number of days when mean T° < -10°C**	*November*	-0.15	0.051	-2.94	< 0.01	- 6.9 days
**Precipitation (mm)**	*May*	0.49	0.18	2.72	< 0.01	+ 22.7 mm
**Snow depth index (mm)**	*January*	-3.95	1.80	-2.19	< 0.05	- 182 mm
	*February*	-4.03	1.98	-2.04	< 0.05	- 185 mm
	*May*	-6.99	2.95	-2.37	< 0.05	- 321 mm
	*October*	-0.52	0.22	-2.43	< 0.05	- 24.1 mm
	*December*	-3.76	1.68	-2.24	< 0.05	- 173 mm

Only the significant changes over time (either positive or negative) of the weather variables over different months are presented in this table. The last column indicates the estimated change over time of each climatic variable over the study period, i.e. from 1970 to 2016.

### Climatic effects on calving date

**Fig 3:**
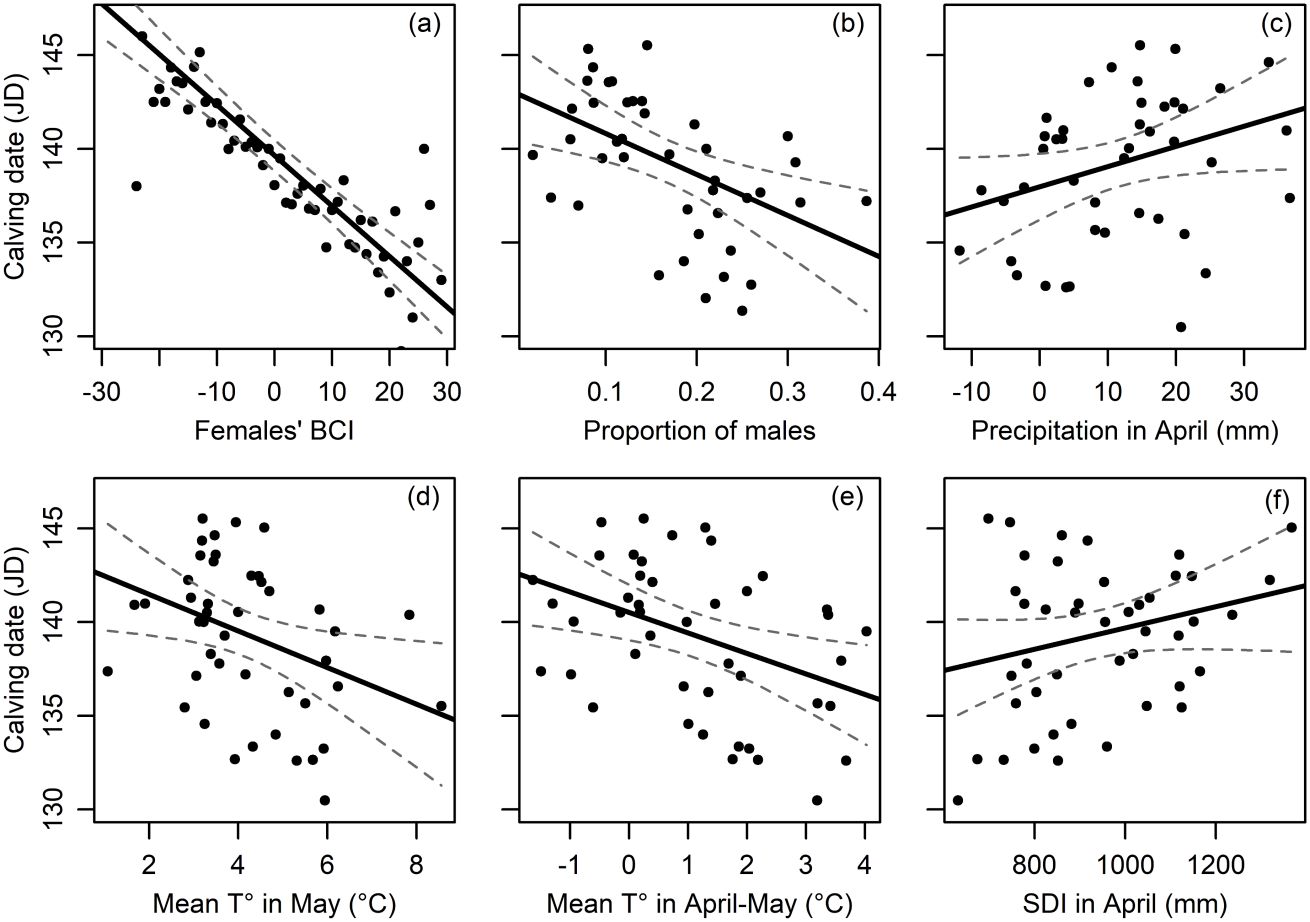
Relationship between reindeer calving date from the Kutuharju field reindeer research station herd from 1970 to 2016 and (a) females’ body condition index (BCI), (b) proportion of males in the herd the precedent breeding season, (c) amount of precipitation in April, (d) mean temperature in May, (e) mean temperature in April-May and (f) snow depth index (SDI) in April. The 95% confidence interval band around the fitted line is provided. The calving date is expressed in Julian day (JD) starting January 1^st^. Each point represents the average value of the predictor variable for a specific calving date.

After comparison of models including local weather variables over different months, three competing models were found to be within 2 AICc of the model with the lowest AICc, i.e. Δ_*i*_ < 2 (see Table 3). These three best models indicated that the most important variables given by the model averaging approach and explaining variation in calving date (in order of effect size) were: the females’ body condition index, the proportion of males in the herd, the amount of precipitation in April, the mean temperature in May, the mean temperature in the period from April to May and the SDI in April (Tables 3 and 4). The three best models showed that calving dates were affected by (1) the female BCI (Table 4, Fig 3a) and (2) the proportion of males in the herd (Table 4, Fig 3b). Accordingly, earlier calving dates were observed with females in better physical condition the year preceding calving (heavier and older; Fig 3a) and in years with a higher proportion of males present in the herd (Fig 3b). The best models also revealed that earlier calving dates were observed following a decreasing amount of precipitation in April (Fig 3c), a warmer climate in May (Fig 3d) and in April-May (Fig 3e), as well as a decreasing snow depth index in April (Fig 3f). These models explained around 44–45% of the variation in calving date.

**Table 3:** AIC table presenting comparative models for calving date of a semi-domesticated reindeer population in Kaamanen, North Finland, including different weather variables over different periods of the year.

Models	Fixed covariates						Calving date				
Rank	♀ BCI	Proportion of males	Mean T°		Precipitation	Snow depth index	AICc	*df*	AICc weights	ΔAICc	*R*^2^
			April-May	May	April	April					
1	×	×		×	×		13894.9	8	0.42	0.0	0.44
2	×	×	×		×		13895.3	8	0.36	0.4	0.45
3	×	×		×		×	13896.2	8	0.22	1.3	0.44

All linear mixed-effects models for calving date included female’s body condition index and proportion of males as fixed effects and female identity and year as random factors. The models presented in the table are the three competing models retained in explaining calving, i.e. with ΔAICc < 2 (see text for details).

**Table 4:** Model-averaged estimates of predictor variables in order of effect size based on the best models in explaining calving date of a semi-domesticated reindeer population in relation to climatic variability in Finnish Lapland.

Variable	Estimate	Unconditional SE	Nbr models	Relative importance	95% CI
Females’ BCI	-1.77	0.19	3	1.00	-2.12, -1.39
Proportion of males	-1.47	0.28	3	1.00	-2.02, -0.91
Precipitation in April	0.93	0.41	2	0.77	0.12, 1.73
Mean T° in May	-1.14	0.43	2	0.63	-1.97, -0.30
Mean T° in April-May	-1.14	0.45	1	0.37	-2.03, -0.26
SDI in April	0.89	0.43	1	0.23	0.03, 1.74

All the competing models were linear mixed-effect models with calving date as our response variable and included *Year* and individual identity as random factors. The parameter estimates are standardized effect sizes and are therefore on a comparable scale. “Nbr models” is the number of models (out of the three best models in Table 3) including that variable.

### Climatic effects on calving synchrony

**Fig 4:**
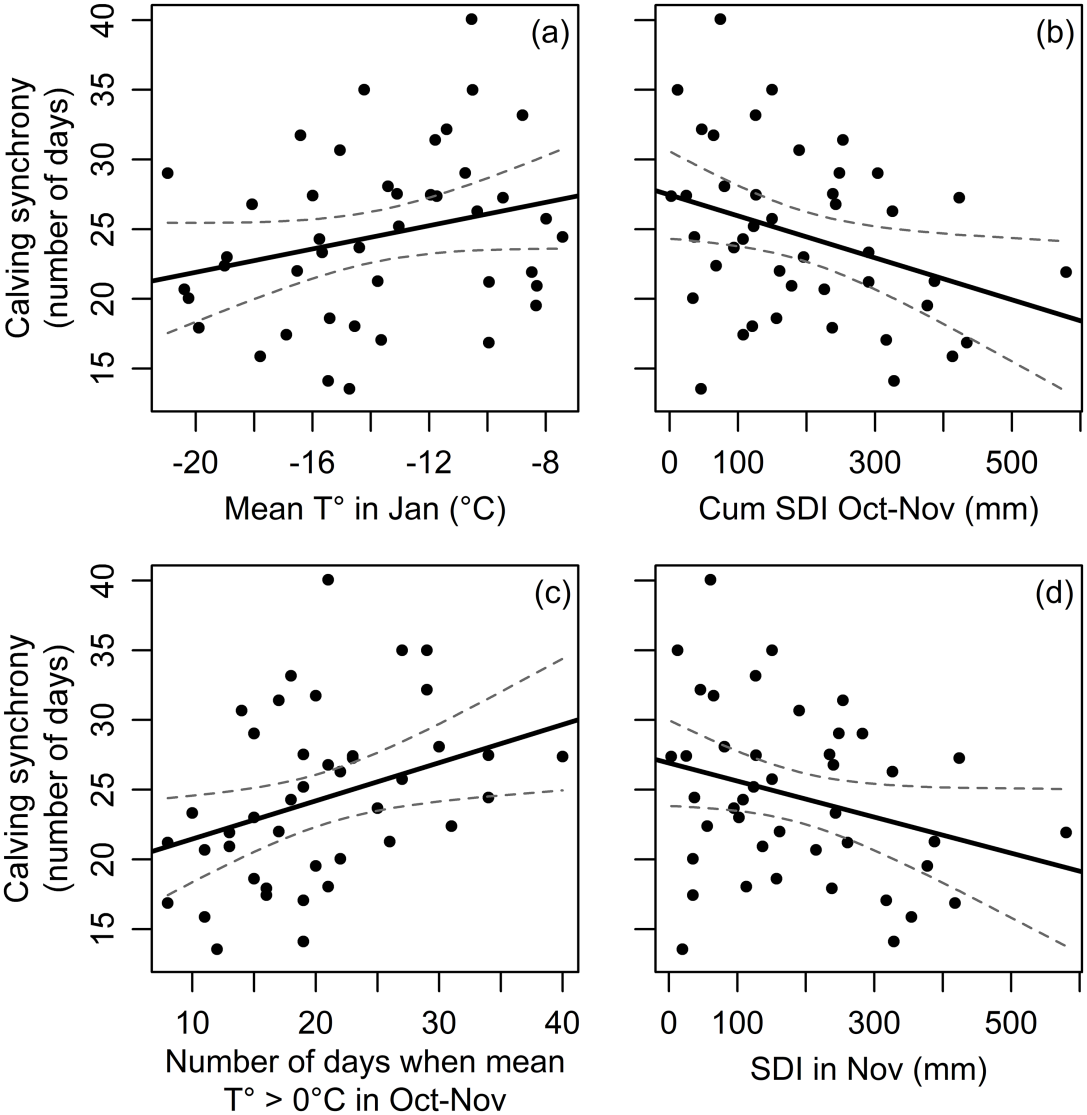
Relationship between calving synchrony of a reindeer population in northern Finland and (a) the mean temperature in January, (b) the cumulative snow depth indexes in the period from October to November, (c) the number of days when mean temperature exceeds 0°C in October-November, and (d) the SDI in November. The 95% confidence interval band around the fitted line is provided. The climatic data from October to November were from the calendar year preceding the year of the calving synchrony whereas the climatic data for January were from the same calendar year as calving synchrony. The calving synchrony was expressed in number of days as reflecting the length of the calving season. The cumulative SDI was the sum of the snow depth indexes for the period of interest.

The model averaging approach applied on the best supported models to explain length of the calving season (see Table 5) indicated that the important variables (whose 95% CI excluded 0) were: the mean temperature in January, the sum of the snow depth indexes from October to November, the number of days when mean temperature exceeded 0°C in October-November, and the SDI in November the precedent calendar year (Table 6). More precisely, a lentghening of the calving season was observed following warmer temperatures in January (Fig 4a), a decreasing cumulative SDI for the period October-November (Fig 4b), a higher number of days when mean temperature exceeds 0°C in October-November (Fig 4c), and a decreasing SDI in November (Fig 4d). The best models also indicated a significant interaction between the mean temperature in January and the average body condition index of females on calving synchrony (Table 6). Apart from this interaction however, both the average BCI of females and the proportion of males were not important in explaing the variation in calving synchrony (Table 4). The competing models explained between 17–23% of the variation in calving synchrony.

**Table 5:** Competing linear models of calving synchrony of a semi-domesticated reindeer population in Kaamanen, North Finland, in relation to local weather variables over different periods of the year.

	Fixed covariates								Calving synchrony				
Rank	♀ BCI	Proportion of males	Mean T°	Number of days when mean T°		Precipitation	Snow depth index		AICc	*df*	AICc weights	ΔAICc	*R*^2^
			Jan	> 0°C in Oct-Nov	< -10°C in Dec	June	Oct-Nov	Nov					
1	(×)	×	(×)				×		276.2	7	0.34	0.0	0.23
2	(×)	×	(×)					×	277.2	7	0.21	1.0	0.22
3	×	×		×	×				277.8	6	0.16	1.6	0.17
4	×	×		×		×			278.0	6	0.14	1.8	0.17
5	(×)	×	(×)			×			278.0	7	0.14	1.8	0.20

All linear models for calving synchrony included female’s body condition index and proportion of males as fixed effects. The climatic conditions in June and in the period from October to December were from the calendar year preceding the calving season whereas the climatic conditions for the month of January were from the same calendar year as the calving season. Variables with the checkmark in brackets mean that the interaction term between both was included in the model. The five models of the table were retained as our best models in explaining calving synchrony, i.e. with ΔAICc < 2 (see text for details).

**Table 6:** Model-averaged estimates of predictor variables in order of effect size based on the best linear models in explaining calving synchrony of a semi-domesticated reindeer population in relation to climatic variability in Finnish Lapland.

Variable	Estimate	Unconditional SE	Number of models	Relative importance	95% CI
Females’ BCI	0.97	1.02	5	1.00	-1.08, 3.02
Proportion of males	-0.76	0.98	5	1.00	-2.73, 1.21
**Mean T° in Jan**	2.13	0.89	3	0.70	**0.34, 3.93**
**Mean T° in Jan X Females’ BCI**	1.97	0.88	3	0.70	**0.19, 3.76**
**Cumulative SDI Oct-Nov**	-2.06	0.83	1	0.34	**-3.75, -0.37**
**Number of days when mean T° > 0°C in Oct-Nov**	2.23	0.87	2	0.30	**0.46, 4.00**
Precipitation in June	-1.77	0.89	2	0.29	-3.58, 0.03
**SDI in Nov**	-1.91	0.84	1	0.21	**-3.62, -0.21**
Number of days when mean T° < -10°C in Dec	-1.93	0.98	1	0.16	-3.93, 0.06

The parameter estimates are standardized effect sizes and are therefore on a comparable scale. “Number of models” is the number of models (out of the five best models in Table 5) including that particular variable. The variables in bold text were assumed important in explaining calving synchrony since their 95% CI excluded the value 0. The symbol “**X**” stands for “interaction”.

## Discussion

### Climatic effects on calving date

The calving season of the semi-domesticated reindeer population of the Kutuharju field reindeer research station in Kaamanen, North Finland has advanced significantly over the last 45 years by almost one week. Eloranta and Nieminen [16] already reported that most of the calving of this same herd occurred on average 19 days between May 10 and 29 and that the peak of calving varied yearly between May the 15^th^ and 25^th^. Similarly, 90% of the caribou calves in North America are born in a brief 2-week period [28]. Therefore, our peak calving date (19-May) matched the previous findings on the same herd [16] but an overall advancement of 6.8 days of the whole calving season represents a consequent change for the calving period in this area. So far, very few studies have highlighted such temporal trends in the reproduction phenology of mammal populations (squirrel [69], red deer [70]). This temporal trend corroborated the overall warming of the spring period from April to May, as well as the reduced snow cover just prior to the births in May observed in the study area the last 45 years. Indeed, earlier calving dates were found following warmer temperatures in April-May, a decreasing amount of precipitation in April (mainly snowfalls at that time of the year) and a reduced snow cover in April the same calendar year. The spring period appears to be critical for ungulate species in northern latitudes, given its influence on the plant growth season pattern [71] and consequently on the food availability during summer. Moreover, late winter/early spring is the most demanding period for reindeer in Arctic since individuals’ body condition and fat reserves to draw upon (reindeer being a capital breeder) are at their lowest point and availability of food is difficult due to hard and thick snow cover [72]. When temperatures rise earlier in spring, the snow starts to melt, and snow free patches will also emerge much earlier, allowing reindeer to easily have access to lichens and dwarf shrubs. Altogether, (1) a better availability of late winter food and a decreasing amount of energy spent in thermoregulation [73,74] and locomotion on snow [75] due to a decreasing amount of snowfalls and a reduced snow cover in April, and (2) an earlier onset of the vegetative growing season [76] and an increased plant biomass observed in the Arctic tundra [77,78] due to warmer temperatures in April-May certainly contributed to increase females’ body condition in late pregnancy. Indeed, further analyses revealed that females’ body weight (a correlate of females’ BCI) had significantly increased over the last 45 years in this population. Such improvement in females’ physical condition will result in mothers having more resources during pregnancy, hence likely advancing the date at which the foetus is mature and resulting in an earlier birth, as compared to years with severe late winter conditions [42]. The significant advancement in calving date could thus be explained by females in better condition giving birth earlier [42–44,79]. The large influence of female body condition on calving date has already been highlighted in numerous studies (bighorn sheep [50], caribou [28,42], elk [45], reindeer [43,44,64]).

An optimal timing of calving will ensure that females have access to a high-quality vegetation (i.e. higher protein content), allowing their calves to be nourished with a high-quality milk [9,14,80] and accelerating the rate of fat accumulation for calves. Moreover, earlier birth dates will (1) provide calves with a longer period of time to sufficiently accumulate fat reserves to survive winter and therefore promoting both their survival and growth, and (2) allow mothers to recover faster from their pregnancy and lactation period and to be in good enough shape to reproduce the next breeding season, promoting both their survival and reproductive success as a result [4,9,81,82]. Therefore, such plastic response is essential for deer species to adapt to climate change by adjusting the period of high energetic requirements (i.e. lactation) with the period of high-quality forage and thereby ensuring offspring survival. Identifying the environmental variables that trigger a plastic response in the reproductive phenology of animal species is thus of primary concern in order to better predict their long term viability. Many species of birds and mammals have already been shown to rely on temperature to match the birth timing with the peak of resource availability [22,83]. In ungulate species, females may adjust their gestation length as a strategy to give birth at the period of the year best suited for offspring survival. Such adjustment of gestation length as part of the reproductive tactic has previously been reported in reindeer [64,84]. On the Isle of Rum, Scotland, warm March temperatures were associated with shorter average gestation lengths in red deer and Clements et al. [85] proposed that high March temperatures could act as a cue to indicate that the optimum birth date is likely to be earlier. The females of the Kutuharju field reindeer research station also seemed to rely on temperature in April-May but also on snow conditions (amount of snowfalls and snow depth index) in April to adjust their gestation length in late pregnancy and consequently calving time within the same year accordingly. Nevertheless, the significant relation between calving dates and mean temperature in April-May do not necessarily mean that females use temperature as a predictive cue for future environmental conditions. This correlation could just be the result of an increased female’s physical condition following improved climatic conditions in late winter/early spring. To demonstrate a cause-effect relationship and whether temperature has a direct signaling effect on seasonal timing, experimental approaches would be necessary but as Caro et al. [83] mentioned, “given the scarcity of experimental approaches investigating this causal effect of temperature, especially in mammals, generalizations are not possible and additional studies are desperately needed”.

### Climatic effects on calving synchrony

The calving synchrony was also affected by an overall warming of the period from August to December as well as a reduced snow cover in winter from December to February reported in the study area since a lengthening of the calving season was observed following an overall warming weather in January and an increasing number of days when mean temperature exceeds 0°C in October-November. The calving synchrony was also weakened by a decreasing snow cover in the period from October to November. Moreover, females with an overall better physical condition (i.e. above the third quantile of the population distribution) delayed their calving dates following a higher number of days when mean temperature exceeds 0°C in October-November (LMM; *b* = 1.31 ± 0.56 SE; 95% CI (0.22, 2.39)) and warmer temperatures in January (LMM; *b* = 1.36 ± 0.60 SE; 95% CI (0.18, 2.53)) whereas females in poor physical condition (i.e. below the first quantile of the population distribution) showed no phenotypic plasticity in their calving dates when facing better climatic conditions in October-November [95% CI = (-1.62, 0.68)] and in January [95% CI = (-0.05, 2.05)]. The lengthening of the calving season following better climatic conditions in October-November and warmer temperatures in January may thus reflect a reduced plasticity among low-quality mothers (young and light females), so that they are not able to respond as quickly as high-quality mothers (older and heavier females) do, to favourable climatic conditions in fall and winter.

The onset of the rut period in deer species has been shown to be mainly triggered by a sudden drop in temperature around the breeding season (around late September/October) which trigger males’ rutting behaviours to start [86–88]. Therefore, a higher number of days when mean temperature exceeds 0°C in October-November, highly correlated with a decreasing snow cover during the same period, would delay the time when males begin to display mating behaviours; resulting in a delay in females’ estrus [89]. Further analyses on a dataset of validated copulation dates (which led to the birth of a calf within the 211–229 days’ time window for gestation lengths reported in this herd [64]) also revealed a delay in copulation dates only for females in poor physical condition in September following a higher number of days when mean temperature exceeds 0°C in October-November (LMM; *b* = 3.06 ± 0.74 SE; 95% CI (1.46, 4.69)). Therefore, the delay in estrus dates following better climatic conditions around the mating time would be more pronounced for females in poor physical condition while females in good condition (old and heavy females) would still be mated earlier [64,89]. As shown in many ungulate species, late copulation dates are also correlated with shorter gestation lengths [64,84,85,90] such as females in poor physical condition not having enough reserves to buffer climatic effects and cope with gestation costs. On the contrary, females in good physical condition would be able to afford the risks of delayed calving dates (as reported above) when climatic conditions in fall are better [91], and afford such the corresponding gestation costs and thus lengthen their gestation lengths [64,85] to improve their calves’ condition at birth, increasing their own reproductive success the following summer as shown in caribou and reindeer [92–94]. The lengthening of the calving season after years with better climatic conditions in October-November (warmer temperatures and a decreased snow cover) would thus be explained by delayed calving dates from females in good physical condition.

The positive relationship between mean temperature in January and calving synchrony was enhanced by females BCI. Indeed, longer calving seasons were observed following warmer temperatures in January contributing to increase females’ physical condition, which in turn delayed their calving dates (as described above). From the mother and offspring’s points of view, delaying calving when climatic conditions in winter are favorable provide selective advantages. A longer gestation length provides (1) the foetus a longer period for growth and development [95,96], (2) a higher offspring’s birthweight [91], ensuring a higher survival probability [35,45,85] and (3) an enhanced fitness for the offspring [93]. A higher offspring’s fitness will certainly mean improved fitness for the mother [93,94]. However, the ability for a female to be plastic requires a higher physical condition to be physiologically able to do so [49,97]. A warming temperature in January would allow pregnant females to spend less energy for thermoregulation [73,74] and reduce the costs of locomotion on snow [75], thus improving their overall physical condition. However, females with an overall higher physical condition would be more able to buffer climatic effects in warmer winters and allocate more resources to growth and development of their foetus [95,96,98], whereas females in poor physical condition would probably prioritize the maintenance of their own body reserves over their foetus’s growth and development [95,96,99]. In warmer winters, only females in good physical condition would be able to delay their calving dates, thereby contributing to a lengthening of the calving season in those years. Such asymmetric response to improved vs. reduced winter conditions has been demonstrated in reindeer as a ‘risk-averse adjustment in reproductive allocation’ [97].

The inter-individual heterogeneity in the response to improved climatic conditions in October-November and January would thus be responsible for the variability in calving synchrony observed in this herd. Understanding what shapes inter-individual heterogeneity in the plasticity of calving date in response to climatic variation would be a natural continuation to this study. We noted that the shift in birth synchrony has occurred in the quasi-absence of predation (20 cases of calves killed by predation out of 2,137 birth dates) so that climatic variability seemed to be one of the main driver shaping calving synchrony in this population. As the competing models explained at best 23% of the variation in calving synchrony, it suggests that other parameters could also be important in explaining variation in the length of calving season (e.g. social, physiological or behavioural cues). Whether phenological changes in calving date and/or calving synchrony have consequences for populations’ recruitment rate and/or females’ reproductive success is a question with contrasted answers among ungulate species. In red deer, Moyes et al. [70] did not find a significant temporal change in either offspring birth weight or offspring first-winter survival whereas parturition date has advanced. On the contrary, Post and Forchhammer [23] found a reduced production and survival of caribou calves following warmer spring temperatures due to a trophic mismatch between the caribou’s timing of calving and onset of the plant growing season in the Low Arctic Greenland. In this semi-domesticated reindeer population, assessing the offspring first-winter survival was not feasible because approximately one third of the calves are slaughtered every fall for meat production. However, calves who survived the summer had earlier birth dates than calves who died either at birth, after one day, one week or later in the summer (LMM; *b* = -1.23 ± 0.37 SE; 95% CI (-1.96, -0.50)). Moreover, Holand et al. (unpublished) have found that both the calving dates and calves’ birth weight of this population are under stabilizing selection with advanced birth dates and increased calves’ birth weights. Whether such selection has consequences in terms of population dynamics and life history traits in this population is yet to be demonstrated but as Gaillard et al. [100] mentioned: “the immature stage, despite a low relative impact on population growth rate compared with the adult stage, may be the critical component of population dynamics of large herbivores”. Furthermore, conditions early in life has been reported to shape lifetime reproductive success [61,101]. Therefore, changes in birth dates and birth weights could have major consequences on population dynamics of ungulate species like reindeer. More studies on this matter are needed with the need to disentangle behavioral/phenotypic plastic responses from microevolutionary responses and the consequences for ungulate populations [102].

### Conclusions

The calving season of the semi-domesticated reindeer population in Kaamanen, northern Finland has occurred earlier following warmer temperatures in April-May, a decreased amount of snowfalls in April and a reduced snow cover in April and has lengthened with a warming weather in January, a higher number of days when mean temperature exceeds 0°C in October-November and a decreasing snow cover in the period from October to November. Such phenological trends have allowed this reindeer population to track at least partially the climatic changes observed in this area. The phenology of many species have changed in response to climate change, particularly at higher latitudes in the Northern Hemisphere but most evidences came from long-term studies of many taxonomic groups other than ungulate species [21,103,104]. As such, this study enhances our understanding of how reproductive phenology of ungulate species would be affected by climate change. That such results on a semi-domesticated reindeer population were observed, where supplemental feeding in harsh winter years could have helped to buffer against climatic conditions, suggests that influence of climatic variation on the reproductive phenology of wild populations might be even stronger. Therefore, more ecological studies linking reproductive phenology of wild populations to climatic variation are needed. While the calving date has already been found to be influenced by temperature and snow conditions [33,35,105], this study is so far the first to highlight an influence of weather variables on calving synchrony in ungulates. In summary, the variability of climatic conditions in the period from October to November and in January seemed important for the variability in females’ plastic response of calving dates to better climatic conditions and as a consequence in shaping calving synchrony at the population level whereas climatic conditions in early spring, just before the calving season, seemed more important in defining the calving dates at the individual level, likely because of its influence on the adjustment of each female’s gestation length.
